# Pharmacological strategies for preventing post-stroke seizures and epilepsy

**DOI:** 10.3389/fneur.2025.1709077

**Published:** 2026-01-08

**Authors:** Yuki Kawamura, Eugen Trinka, Terence J. Quinn, Hedley C. A. Emsley, Johan Zelano, Tomotaka Tanaka, Masafumi Ihara, Lauren H. Sansing, David S. Liebeskind, Nishant K. Mishra

**Affiliations:** 1 Department of Neurology, Yale School of Medicine, New Haven, CT, United States; 2 School of Clinical Medicine, University of Cambridge, Cambridge, United Kingdom; 3 Department of Neurology, Center for Cognitive Neuroscience, Christian Doppler University Hospital, Paracelsus Medical University, Member of the ERN EpiCARE, Salzburg, Austria; 4 Neuroscience Institute, Center for Cognitive Neuroscience, Christian Doppler University Hospital, Paracelsus Medical University, Salzburg, Austria; 5 Karl Landsteiner Institute for Clinical Neuroscience, Salzburg, Austria; 6 School of Cardiovascular and Metabolic Health, University of Glasgow, Glasgow, United Kingdom; 7 Lancaster Medical School, Lancaster University, Lancaster, United Kingdom; 8 Institute of Neuroscience and Physiology, Department of Clinical Neuroscience, Sahlgrenska Academy, University of Gothenburg, Gothenburg, Sweden; 9 Wallenberg Center of Molecular and Translational Medicine, University of Gothenburg, Gothenburg, Sweden; 10 Department of Neurology, Sahlgrenska University Hospital, Member of the ERN EpiCARE, Gothenburg, Sweden; 11 Department of Neurology, National Cerebral and Cardiovascular Center, Osaka, Japan; 12 Department of Neurology, Faculty of Medicine, Shimane University, Izumo, Japan; 13 Department of Neurology, University of California Los Angeles, Los Angeles, CA, United States

**Keywords:** stroke, post-stroke epilepsy, seizures, stroke outcome, cerebral haemorrhage, antiseizure medication

## Abstract

Stroke is the most common cause of new-onset seizures and epilepsy in the older population, which is associated with increased morbidity and mortality. Post-stroke seizures (PSS) are traditionally divided into early and late seizures, occurring before and after 7 days post-stroke, respectively. A single late seizure is sufficient to diagnose post-stroke epilepsy. This narrative review discusses approaches to diagnosing and treating PSS, as well as the various pharmacological agents available. Although current evidence is limited, we suggest that levetiracetam and lamotrigine may be preferred agents for preventing acute seizure recurrence. Statins, GLP-1 agonists, eslicarbazepine, perampanel, and losartan have not been evaluated yet and need further study on their ability to prevent first-time seizures in stroke patients. While clinical trials of antiseizure medications can be costly, further research into biomarkers of epileptogenesis could facilitate more feasible clinical trials to enhance the evidence base for antiseizure medications in post-stroke seizures and epilepsy.

## 1 Introduction

Cerebrovascular disease accounts for nearly 50 % of new-onset seizures and epilepsy in individuals over 65 years of age (1). Post-stroke seizures (PSS) are linked with increased morbidity, mortality, and cognitive impairment compared to those without. Early recognition and treatment of PSS are therefore crucial steps in reducing the disease burden of stroke, but existing literature on its pharmacological management remains limited (2–4). Importantly, current therapies for PSS usually control seizures but do not address the underlying disease process, and no treatment has yet been demonstrated to prevent the development of epilepsy after a stroke in humans (5, 6). There is a critical need to develop anti-epileptogenesis strategies for the primary prevention of PSS that target the underlying disease mechanism, as well as to identify effective medications for preventing seizure recurrence (7). In this narrative review, we summarise the current evidence regarding diagnostic and treatment approaches for PSS, along with the effectiveness, safety, and drug–drug interactions of ASMs.

## 2 Definitions and diagnostic approach

Key terms and definitions are described in Table 1. PSS can be subdivided, depending on the time before onset, into early post-stroke seizures (EPSS), which occur less than 7 days after stroke, and late post-stroke seizures (LPSS), which occur after a week. Unlike EPSS, which are considered provoked seizures due to toxic or metabolic effects of stroke, LPSS can be considered as unprovoked seizures (8). LPSS are associated with a higher risk of seizure recurrence than EPSS (10-year recurrence risk of 65% vs. up to 33%, respectively (9–11)). The International League Against Epilepsy (ILAE) definition of epilepsy includes “one unprovoked (or reflex) seizure and a probability of further seizures similar to the general recurrence risk (at least 60%) after two unprovoked seizures, occurring over the next 10 years” (12); hence, one LPSS is sufficient to diagnose *post-stroke epilepsy (PSE)*.

**Table 1:** Key terms and definitions.

Term	Definition
Post-stroke seizures (PSS)	Seizures observed in patients after a stroke
Acute symptomatic seizures	Seizures occurring at time of or close temporal association with a brain insult^1^
Early Post-Stroke Seizures (EPSS)	Seizures observed in patients within 7 days of a stroke
Late Post-Stroke Seizures (LPSS)	Seizures observed in patients after 7 days post-stroke
Post-Stroke Epilepsy (PSE)	Unprovoked late post-stroke seizures
Primary prevention	Administration of pharmacotherapy to prevent epileptogenic abnormalities
Primary prophylaxis	Administration of pharmacotherapy to prevent the incidence of seizures, without necessarily modifying the underlying epileptogenic abnormality
Secondary prophylaxis	Administration of pharmacotherapy to prevent recurrence of seizures in patients who have already had a post-stroke seizure, without necessarily modifying the underlying epileptogenic abnormality

^1^ Beghi et al. (137).

Current clinical definitions of PSS in effect utilise time between the stroke and seizure as a proxy to infer whether the seizure episode is due to direct injury from stroke (EPSS) or the likely presence of underlying epileptogenic changes (LPSS). However, the temporal course of epileptogenesis is likely to be more nuanced (13). Indeed, a study demonstrated that patients with PSS 4–7 days after a stroke are more likely to develop PSE compared to those with seizures within 3 days (14), suggesting that changes conferring longer term seizure risk can commence even within the first week. Interestingly, a more recent multicentre study on 4,552 patients found that seizures on the day of the stroke was associated with a higher risk of PSE compared to EPSS after 1 day (15), again implying pathophysiological changes occurring very early after stroke onset can influence the risk of PSE. In particular, patients with focal to bilateral tonic–clonic seizures on the day of stroke had a 69% 10-year risk of LPSS, exceeding the risk threshold used in the ILAE definition of epilepsy, although they would not qualify as having epilepsy since EPSS are currently considered as acute symptomatic (or provoked) seizures. Towards the other end of the time spectrum of late seizures, new-onset seizures occurring more than 2 years after stroke have a lower recurrence risk than late seizures occurring within the first 2 years (16). Together, these findings demonstrate that time from stroke to first seizure fails to capture the full complexity of epileptogenic changes after a stroke. As most seizure recurrences after EPSS occur within 1–2 years (17), with risk declining sharply thereafter, we need more dynamic tools, such as the Chance of an Occurrence of a Seizure in the Next Year (COSY) and validated prognostic models, such as the SELeCT 2.0 (18) and CAVE scores (19).

Multimodal approaches integrating EEG monitoring can further boost predictive capability and have been incorporated in scoring systems such as the SeLECT-EEG score for risk prediction after ischaemic stroke (20). These integrative approaches can aid rationalised PSE treatment, as demonstrated in a decision analysis study which found that using a risk-guided approach to pharmacological treatment guided by an EEG-based risk stratification tool can improve outcomes in certain clinical scenarios (21). Interictal epileptiform discharges in PSE patients also independently predict recurrence of seizures (22), lending further support to the utility of tools that are able to capture richer physiological parameters suggestive of epileptogenesis.

**Figure 1:**
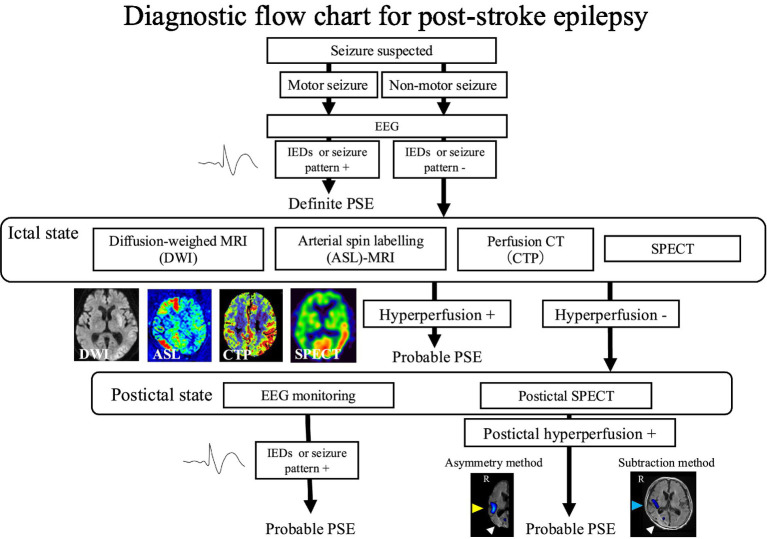
Schematic of clinical approach to post-stroke seizures. Used with kind permissions of Drs Tomotaka Tanaka, Masafumi Ihara, and Kazuki Fukuma (IED: Interictal epileptic discharges).

In view of these findings, a tissue-based (in contrast to time-based) approach to diagnosing PSE has recently been proposed (23). While there is currently no consensus for such an approach, a proposed tissue-based diagnostic approach to PSE starts with the assessment of epileptiform discharges or active biological biomarkers. Following clinical evaluation, the initial investigation consists of using an electroencephalogram (EEG) to assess for the presence of interictal discharges and seizure patterns (Figure 1). The presence of hallmark epileptic features on EEG leads to the diagnosis of PSE. Alternatively, biomarkers have been proposed to assess epileptogenesis (5). Although reliable biomarkers have not yet been established, once discovered, they will likely contribute to tissue-based PSE definitions (23). Status epilepticus in EPSS has been identified as a risk factor for PSE (15, 24) and could be a potential candidate for a biomarker, but it is yet unclear whether this indicates actual epileptogenicity or is simply associated with a higher risk of epileptogenesis. In the absence of hallmark EEG features or biomarkers, hyperperfusion in the affected regions identified by imaging modalities such as diffusion-weighted imaging (DWI), CT perfusion (CTP), arterial spin labelling (ASL)-MRI, or SPECT (25, 26) during the ictal phase can indicate probable PSE. If ictal imaging findings do not indicate hyperperfusion, the presence of hallmark seizure features on postictal EEG or hypoperfusion on postictal SPECT can point towards a probable diagnosis of PSE. While neuroimaging can provide valuable insights into underlying epileptogenic changes, hyperperfusion changes are not always concordant with EEG-based localisation (27, 28), and ambiguity also remains whether other imaging findings associated with epilepsy are specific signs of epileptogenesis or artefacts of the initial insult (29, 30). Diagnosis of PSE currently remains clinical, and further refinement of imaging criteria are needed before they can be used as reliable markers of epilepsy.

## 3 Pathophysiology

**Figure 2:**
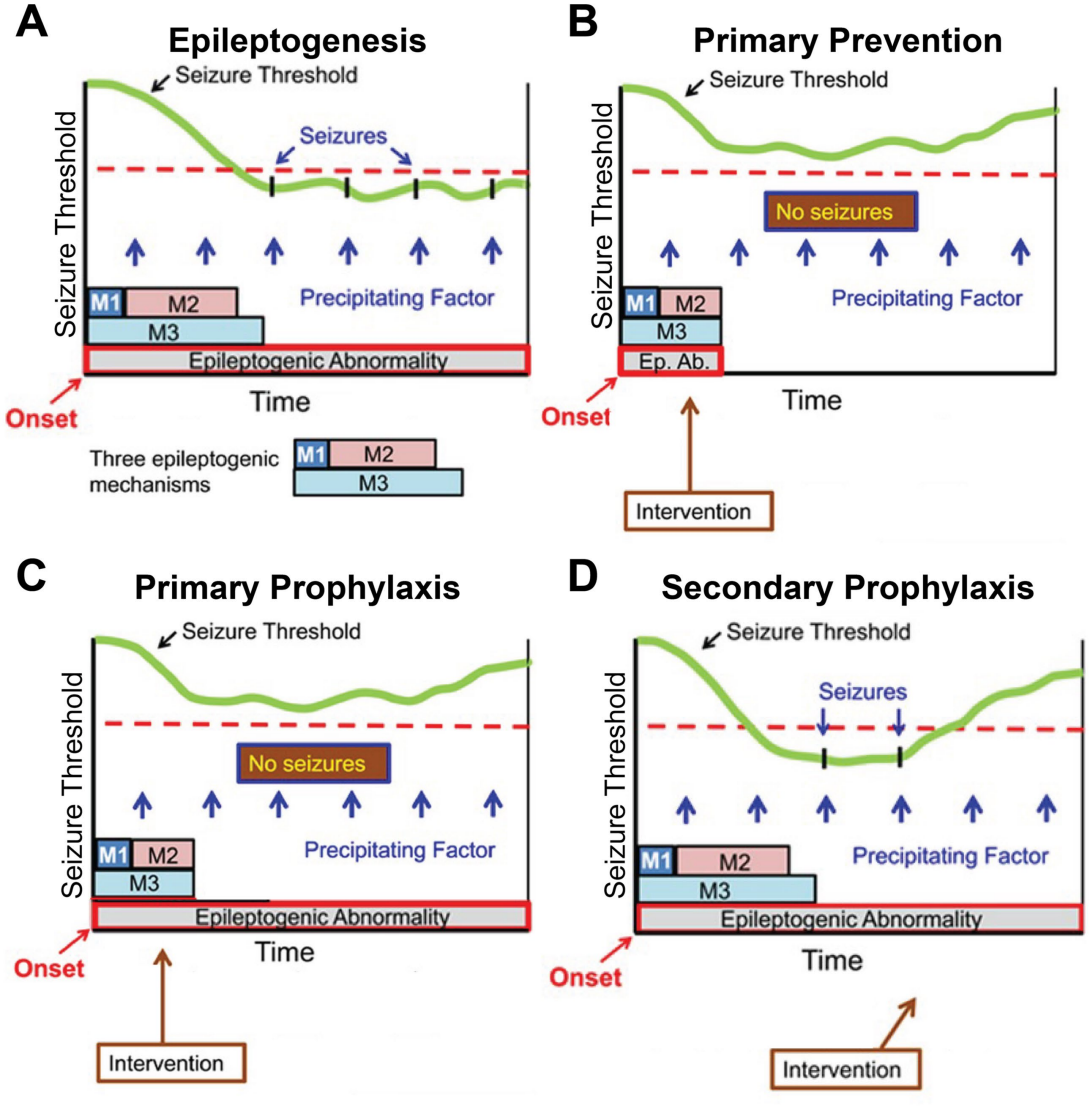
Schema of epileptogenesis **(A)**, primary prevention **(B)**, primary prophylaxis **(C)**, and secondary prophylaxis of seizures **(D)**. Adapted from Engel et al. (11) with permission from the publisher. Epileptogenesis is considered a cascade of several pathogenic mechanisms (illustrated as M1, M2, and M3) combining to create an epileptogenic abnormality. Once the seizure threshold (in light green) goes below a certain level (dotted line), seizures can occur spontaneously or in response to precipitating factors.

The process of epileptogenesis can be considered as a cascade of pathobiological processes which reduces the seizure threshold (illustrated in green in Figure 2) to a level at which seizures can occur in response to precipitating factors (Figure 2A). The purpose of primary prevention is to prevent epileptogenic processes from progressing and maintain a seizure threshold high enough to prevent spontaneous seizures from occurring (Figure 2B) (31). An ideal approach would be to eliminate the effects of epileptogenic mechanisms so that an underlying abnormality does not develop. Primary and secondary prophylaxis also aim to increase the seizure threshold so that no seizures occur for the first time or recur after an episode, respectively, but without necessarily modifying the underlying epileptogenic abnormality (Figures 2C,D).

EPSS and LPSS are widely considered to have distinct pathophysiological mechanisms, albeit with imperfect separation and some evidence, as alluded to above, of overlap. The pathogenesis of EPSS has generally been attributed to excitotoxicity due to ion channel dysfunction, reduction of seizure threshold due to attenuation of GABAergic signalling, elevated cortisol concentration causing neurotoxicity, and haemosiderin deposits leading to increased oxidative stress (32). Conversely, the pathogenesis of LPSS is associated with gliotic scarring secondary to persistent inflammation, epileptogenic pathways that follow blood–brain barrier disruption, and related changes to neuronal networks (33, 34). In regions with blood–brain barrier disruption, blood-derived albumin can bind to the transforming growth factor-*β* receptor and reduce astrocytes’ uptake of potassium ions and glutamate, resulting in a lower seizure threshold (32).

In addition to the differences in pathophysiology, EPSS and LPSS are also associated with different risk factors for recurrence. Status epilepticus and male sex were associated with an increased risk of recurrence in patients with EPSS, whereas younger age was associated with increased seizure recurrence in those with LPSS (10, 35). The epidemiology of PSS is also affected by the inciting aetiology. PSS are more common in intracerebral (ICH) or subarachnoid haemorrhage (SAH) in comparison to ischaemic stroke (incidence rate of 10–16% vs. 3–6% in the acute phase) (36, 37). Haemosiderin deposition, blood–brain barrier disruption, and cortical superficial siderosis are strongly associated with seizure induction (23), which is consistent with the higher PSS risk observed in patients with ICH, SAH or ischaemic stroke with haemorrhagic transformation (38). Hence, both the timing of PSS and inciting stroke subtype are likely to be relevant considerations in pathogenic mechanisms targeted in pharmacotherapy. The current review includes a summary of studies performed specifically for ischaemic stroke or ICH and SAH cohorts.

## 4 Pharmacology

### 4.1 ASMs for primary and secondary prophylaxis

#### 4.1.1 First-generation ASMs

##### 4.1.1.1 Clinical effectiveness

First-generation ASMs include agents that act on voltage-gated sodium channels, such as phenytoin and carbamazepine, agents that act on GABA_A_ receptors, such as phenobarbital and diazepines, and agents such as valproic acid whose mechanisms have not yet been fully elucidated (39).

A Cochrane Review on ASMs for primary PSS prophylaxis published in 2022 identified two randomised control trials, both of which involved first-generation ASMs (valproic acid and diazepam). It concluded that ASMs were not shown to be effective in primary prophylaxis of PSS (40). The first trial administered valproic acid orally with an initial loading dose of 400 mg twice daily, then adjusted to maintain a mild therapeutic dose (50-100 μg/dL) to 72 patients. The study failed to find a significant difference in the incidence of EPSS (2.7% vs. 11.1%, treatment vs. placebo, respectively) and LPSS (16.6% vs. 11.1%) between valproic acid and placebo, but observed better neurological outcomes after 12 months in the treatment group (41). The second trial compared treatment with diazepam 10 mg rectally as soon as possible after stroke, followed by 12-hourly therapy for 3 days or earlier until discharge with a placebo in 784 patients. The trial reported no significant difference in seizure occurrence in either group (1.5 vs. 3.3%, treatment vs. placebo, respectively), but the results achieved significance in favour of treatment if restricted to patients with cortical infarcts in the anterior circulation (42).

Neither study reported results on secondary prophylaxis of EPSS or LPSS. A retrospective cohort study of 3,622 people with PSS based on the national insurance database in Taiwan demonstrated that hospitalisation for seizure recurrence was highest in patients taking phenytoin and higher in those taking valproic acid and carbamazepine in comparison to newer ASMs (43). In summary, there is limited evidence to suggest that first-generation ASMs are effective for primary and secondary prophylaxis of PSS.

##### 4.1.1.2 Safety/tolerance

Valproic acid has been associated with thrombocytopaenia in 5–18% of general adult patient cohorts, manifesting in prolonged bleeding time, abnormality in platelet laboratory values, or petechiae (44). This effect has been replicated *ex vivo* in blood samples from patients treated with valproic acid, which resulted in decreased platelet aggregation (45) as well as in blood collected from an animal model exposed to valproic acid and haemorrhagic shock, which resulted in significantly reduced platelet aggregation, clot strength, and clot formation rate (46). Modelling the risk of thrombocytopaenia demonstrated that female sex, high trough free valproic acid levels, and baseline platelet counts are associated with increased risk of thrombocytopaenia in patients administered valproic acid (47). Potential effects on coagulation properties could complicate prescription of valproic acid in conjunction with anticoagulants in stroke survivors with high clotting risk. In addition, a particular concern in older patients is the risk of valproate encephalopathy, a syndrome not yet fully understood that can include cognitive decline, brain atrophy, tremor/parkinsonism (16, 48).

Some first-generation ASMs have relatively higher risks for foetal malformations when used in pregnancy. Valproic acid is associated with major malformations and a 1–3% risk of neural tube defects (49), and is therefore contraindicated in women and girls of childbearing potential, with avoidance also advised in men (MHRA, CHM advice), although the strength of advice can vary, such as in Japan (50). Phenytoin is associated with foetal hydantoin syndrome in around 11% of children exposed *in utero*, with an additional 30% of exposed children expressing some of the pathological features (51).

##### 4.1.1.3 Drug–drug interactions

Valproic acid is involved in several drug–drug interactions by inhibiting the metabolism of other medications. For example, valproic acid increases the half-life of lamotrigine two- to three-fold and is also known to increase serum concentrations of phenobarbital, phenytoin, carbamazepine, and rufinamide (52). Dose adjustments might be necessary when valproic acid is added to the pharmacological regimen to minimise potential side effects.

Many first-generation ASMs can alter the pharmacokinetics of direct oral anticoagulants (DOACs) through induction of cytochrome enzymes and P-glycoprotein. This is a relevant consideration given that 1.4% of patients on DOACs also take cytochrome enzyme-inducing drugs, mostly for seizure treatment (53). The reported effects of enzyme inducers such as phenytoin and carbamazepine on bleeding or thromboembolic risk are varied in literature. Multiple studies reported reduced plasma DOAC levels (54, 55) and increased risk of stroke (56) in patients concurrently prescribed enzyme-inducing ASMs and DOACs. On the other hand, a retrospective cohort study in Taiwan concluded that concomitant use of phenytoin with dabigatran or rivaroxaban led to increased risk of bleeding (57). The latter result appears paradoxical considering that phenytoin is a CYP3A4 and P-glycoprotein inducer which should normally lead to reduced plasma DOAC levels (58, 59). Clinicians prescribing DOACs with first-generation ASMs, many of which are enzyme inducers, should be cognizant of this interaction to prevent anticoagulant treatment failure. Other cardiovascular medications whose concentrations can be lowered by inducers of CYP3A4 include calcium channel blockers such as amlodipine as well as atorvastatin and simvastatin (60, 61).

#### 4.1.2 Second-generation ASMs

##### 4.1.2.1 Clinical effectiveness

Second-generation ASMs include agents such as lamotrigine and topiramate which primarily act on voltage-gated sodium channels; levetiracetam, which appears to act on SV2A receptors; and gabapentin, which primarily acts on high-voltage-associated calcium channels (3, 39).

The efficacy of levetiracetam for primary prophylaxis was investigated in the *Prevention of Epileptic seizures at the Acute phase of intraCerebral Haemorrhage* (PEACH) trial (62) which saw a significant reduction in the number of clinical or electrographic EPSS after supratentorial intracerebral haemorrhage in the treatment group, although the study was interrupted early and thus underpowered (*n* = 50).

Comparison studies generally demonstrate similar effectiveness for secondary prophylaxis but better tolerability in second generation ASMs in comparison with first-generation ASMs. A randomised control trial comparing the effects of levetiracetam (titrated up to 500 mg twice daily) and carbamazepine (titrated up to 300 mg twice daily) in 128 patients with LPSS demonstrated a nonsignificant trend towards a higher seizure-free ratio for patients taking levetiracetam (94% vs. 85%, *p* = 0.08) (63). Another randomised control trial included 64 patients with EPSS or LPSS and compared the effects of lamotrigine (titrated up to 100 mg twice daily) versus carbamazepine (titrated up to 300 mg daily) which demonstrated a nonsignificant trend towards decrease in seizure recurrence within 12 months in the lamotrigine group (64). Of note, levetiracetam and lamotrigine had no significant difference in seizure freedom according to a network meta-analysis based on these studies (65). A more recent network meta-analysis comparing 13 antiseizure medications including both first- and second-generation ASMs in stroke (66) suggested that levetiracetam was among the pharmaceutical agents with the lowest seizure recurrences in comparison to other agents.

##### 4.1.2.2 Safety/tolerability

Levetiracetam and lamotrigine were shown to have the best side-effect profile compared to first-generation agents and with other pharmacological regimens (66) through network meta-analysis, with the most common side effects being fatigue, somnolence, headache, and dizziness (67, 68). Both medications are well-tolerated, with lamotrigine being discontinued due to adverse effects in 9–16% of epilepsy patients (69, 70) and levetiracetam in 15% of patients in a LPSS prospective cohort (71). Notable adverse effects of levetiracetam are behavioural or psychiatric symptoms, which can be observed in 7–14% of patients (72, 73) and should be considered when prescribing to patients with psychiatric comorbidities or at higher risk of psychiatric symptoms. Given that depression and irritability are common in poststroke populations (61 and 33%, respectively, in one study) (74), it is important to consider this adverse effect. Brivaracetam, an analogue of levetiracetam, is a third-generation medication associated with an improvement in behavioural adverse effects (75) and might be a preferable option for patients at higher risk of psychiatric symptoms.

Rarer side effects of levetiracetam include haematological side effects such as thrombocytopaenia, which is usually transient. A causal relationship for thrombocytopaenia was established only in around 0.1% of patients in a retrospective study in a general inpatient cohort (76), and thus this adverse effect appears to be rare. In addition, although it has been posited that levetiracetam could cause platelet dysfunction, this has not been replicated in assays on healthy volunteers in a double-blind crossover study (45).

Lamotrigine and levetiracetam were not associated with an increased risk of major congenital malformations compared to patients who were not exposed to ASMs (77). Indeed, multiple studies failed to find any major congenital malformations in children exposed *in utero* and a study from the North American registry finding a malformation rate of 2.03% after monotherapy, which is lower that of most other ASMs (78).

##### 4.1.2.3 Drug–drug interactions

Second-generation ASMs generally have less serious drug–drug interactions in comparison to first-generation ASMs (79) as they do not induce metabolic enzymes to the same extent. Levetiracetam has a low risk of drug–drug interactions since it mostly circulates unbound to proteins (80) and is primarily excreted renally (75%) (61). Levetiracetam induces P-glycoprotein but not CYP3A4 *in vitro* (81, 82), but does not affect the serum concentration of digoxin, a P-glycoprotein substrate, in healthy volunteers after repeated administration (83). Lamotrigine is metabolised through glucuronidation by UGA1A4 and does not generally interact with other drug-metabolising enzymes. Its apparent clearance increases in the presence of combined oral contraceptives, likely due to the induction of the UDP-glucuronidase system (84). In contrast, lamotrigine did not affect the pharmacokinetics of combined oral contraceptives but reduced the maximum concentration and AUC of levonorgestrel at a clinically insignificant level and much less than phenytoin, carbamazepine, or oxcarbazepine (84). Dose monitoring and adjustment might be prudent for patients who co-administer lamotrigine, oral contraceptives, and valproic acid (see discussion in 4.1.1) to prevent breakthrough seizures. Despite the absence of metabolic enzyme induction, levetiracetam was linked to a higher risk of systemic embolic events and stroke in DOAC-treated patients in some studies (56, 85, 86), although others found no effect (87–89). Other mechanisms, such as pharmacodynamic interactions, could account for such effects but remain unknown, and it is unclear whether the increased stroke risk results from DOAC treatment failure or an intrinsic effect of levetiracetam, as discussed in the previous section (63).

#### 4.1.3 Third-generation ASMs

##### 4.1.3.1 Clinical effectiveness

Third-generation ASMs, such as eslicarbazepine acetate, lacosamide, and perampanel, are being investigated for primary or secondary prophylaxis of post-stroke seizures but there are a limited number of published studies (79, 90, 91). Perampanel was associated with >50% reduction in seizure frequency in 69.1% of LPSS patients after 3 months (92) and 66.7% in another study, which increased to 83.9% after 12 months (93). Eslicarbazepine acetate achieved >50% seizure reduction in 72.9% of patients after 12 months (94) and lacosamide in 80% of patients (95). Eslicarbazepine acetate and lacosamide were also among those with the lowest seizure recurrence in post-stroke cohorts in a network meta-analysis of 13 ASMs (66).

##### 4.1.3.2 Safety/tolerability

Lacosamide and eslicarbazepine acetate appear to have high tolerability, with retention rates being 91.7 and 90.7% at 12 months respectively, compared to 82.0% for lamotrigine and 77.8% for levetiracetam in a retrospective post-stroke cohort study (96). Perampanel had a similar retention rate of around 92.8–94.8% after 3 months in a post-stroke cohort (92, 93). Common side effects for lacosamide and eslicarbazepine acetate are identical to other sodium channel blockers, such as dizziness, diplopia, vomiting, somnolence, and fatigue (97). Aside from more common adverse effects, perampanel is also associated with a black box warning for psychiatric adverse effects such as aggression, hostility, and suicidal ideation, which are more frequent at 8 or 12 mg compared to placebo with a dose–response relationship (98). Although the evidence is mixed regarding suicidal ideation (97), this side effect is more important in the context of stroke survivors, who have an increased risk of suicide (99).

##### 4.1.3.3 Drug–drug interactions

Eslicarbazepine acetate has been shown to reduce the maximum plasma concentration of simvastatin by 38.88% and AUC by 50.43% when co-administered, likely through the induction of CYP3A4 (100). Perampanel has only weak enzyme inducing properties (61) and lacosamide is not a CYP inducer (101); hence, both drugs minimally affect the concentration of other drugs, although the efficacy of perampanel is significantly reduced in the presence of strong enzyme inducers (102). The role of possible P-glycoprotein induction has also not been investigated. Hence, careful monitoring and appropriate adjustment of statin dose would be prudent for patients on eslicarbazepine acetate, especially if they require high intensity lipid lowering therapy. In addition, avoiding perampanel as add-on therapy to strong enzyme inducers is likely to result in better seizure control.

### 4.2 Medications for primary prevention of PSS

#### 4.2.1 Statins

##### 4.2.1.1 Clinical effectiveness

Several studies have associated statins with favourable outcomes in PSS for primary prevention. A meta-analysis (103) demonstrated that post-stroke statin use was associated with lower incidence of EPSS and LPSS regardless of whether the stroke was haemorrhagic or ischaemic. Notably, the study demonstrated that pre-stroke statin use was not associated with reduced risk of EPSS or LPSS. While effective for both EPSS and LPSS, statins were shown to be especially effective for the prevention of EPSS in another meta-analysis (104). Lipophilic statins and moderate to high doses per 2013 ACC/AHA guidelines were associated with a reduced risk of LPSS in a retrospective intracerebral haemorrhage cohort study (105). Interestingly, statins were also associated with antiepileptogenic effects in a variety of animal models and in clinical studies in multiple cohorts with various mechanisms of brain injury, including brain tumours, radiotherapy, and coronary revascularisation in older patients, which suggest their broad antiepileptogenic properties (106). Taken together, the administration of statins after stroke appears to lower the risk of PSS and therefore makes statin a viable agent for primary PSS prevention in addition to its role in managing dyslipidaemia. However, prospective trials are lacking and would be important in establishing the evidence base for the use of statins to prevent PSE.

##### 4.2.1.2 Safety/tolerability

Statins have a good safety profile and were not associated with an increased risk of serious adverse events in a prospective cohort study investigating the effect of high-intensity statins on TIA and stroke patients (107). Persistent elevation of alanine aminotransferase and aspartate aminotransferase was seen more frequently in patients taking statins, although there were no cases of liver failure in this study. Statins are contraindicated in pregnancy and lactation as it is not possible to prove that statins are safe in pregnancy (108).

The discussion on the adverse effects of statin use in stroke patients centres on a possible increase in haemorrhage when statins are used in stroke populations. Indeed, a meta-analysis based on subgroup analyses of the SPARCL and HPS trials (109) showed an increased risk of haemorrhagic stroke when statins were used for secondary prevention in stroke patients. However, a more recent meta-analysis (110) performed on ischaemic stroke patients demonstrated that new statin use was associated with a reduced risk of early intracranial haemorrhage (occurring within 2 weeks of stroke) and did not affect risk of intracranial haemorrhage overall. Trials such as the Statin Use in Intracerebral Haemorrhage Patients (SATURN) trial (111) are ongoing to investigate haemorrhage risk in post-ICH populations. The AHA scientific statement on statin use (108) concludes that while an increased risk of haemorrhage is possible, the absolute risk is small and the benefit in reducing overall stroke and other vascular events outweighs this risk.

##### 4.2.1.3 Drug–drug interactions

Statins are metabolised in many steps, which accounts for their complex drug–drug interactions. Statins are first absorbed in the gut wall, during which P-glycoproteins reduce their concentration in the portal circulation; they are then taken up by hepatic cells by organic anion transporting polypeptide 1B1 (OATP1B1) and subsequently metabolised by CYP enzymes and undergo glucuronidation (112). Agents which induce or inhibit these transporters and metabolic enzymes affect the pharmacokinetics of statins and vice versa, leading to drug–drug interactions.

Most statins (atorvastatin, cerivastatin, simvastatin, lovastatin) are metabolised in the liver by CYP3A4 enzymes and fluvastatin by CYP2C9 (60). Hence, inhibitors of CYP enzymes including cardiovascular drugs such as calcium channel blockers (nifedipine, felodipine, mibefradil, diltiazem, verapamil), other antiarrhythmics (lidocaine) as well as protease inhibitors (indinavir, ritonavir, nelfinavir, saquinavir, etc.), macrolides, and azoles (ketoconazole, itraconazole) are among the drugs which can cause increased plasma concentration of statins and therefore precipitate adverse effects such as myopathy. Conversely, CYP inducers such as troglitazone and rifampicin can reduce plasma doses and require higher doses of statins. Grapefruit juice contains bergamottin, which is an inhibitor of both CYP3A4 and OAT (112), and should be avoided in patients taking statins.

Statins have also been shown to potentiate the effect of warfarin in some cases, and although the effects are usually clinically negligible, there have been reports of more marked bleeding in a small number of patients (60). Hence, careful monitoring of the INR is needed to adjust warfarin dose to appropriate levels if needed.

#### 4.2.2 ARBs

Angiotensin receptors are upregulated in rat models of epilepsy especially in the hippocampus, and ARBs appear to exert anti-seizure effects through a mechanism partially independent of lowering blood pressure (113). Indeed, TGF-*β* signalling driven by extravasated albumin after blood–brain barrier compromise is sufficient to induce epileptiform activity, and this signalling is attenuated by the application of losartan (114). A recent retrospective cohort study involving over 2 million patients has shown that angiotensin receptor blockers (ARBs) are associated with reduced incidence of epilepsy (115), although the study was not specifically in a post-stroke setting. Censoring the patients based on stroke incidence increased the magnitude of the protective effect of ARBs, suggesting that ARBs exert their protective effect through other mechanisms than simply reducing the incidence of stroke. However, this association was not seen in patients with preexisting stroke in this study. In a different retrospective cohort study in Taiwan, angiotensin converting enzyme (ACE) inhibitors and ARB use were associated with longer PSE-free survival period especially in people under 85 (116). Further studies on post-stroke cohorts are warranted to reach more definitive conclusions about the potential effects of ARBs on PSS.

#### 4.2.3 Diuretics

Anti-seizure effects of diuretics have been reported from studies investigating the effects of diuretics on neuronal activity in experimental animal models of seizures as well as in patients undergoing resection of epileptic foci (117). The mechanism of action has been posited to be due to carbonic anhydrase inhibition and attenuation of chloride current, which alters glutamate packaging (118). However, while there is evidence to suggest antiseizure effects for diuretics such as hydrochlorothiazide, chlorothiazide, indapamide, furosemide, bumetanide, and acetazolamide, some diuretics such as cyclothiazide and theobromine can be proconvulsant, and others can cause hyponatraemia which can promote seizures when combined with ASMs such as carbamazepine, oxcarbazepine, and eslicarbazepine acetate (117). There is no evidence regarding the use diuretics for primary prevention of PSS.

#### 4.2.4 GLP-1 agonists

A recent meta-analysis on data from 27 randomised clinical trials (119) demonstrated that GLP-1 agonists, but not DPP-4 inhibitors or SGLT2 inhibitors, were associated with reduced incidence of seizures as well as a combined outcome of seizure and epilepsy, although this analysis was not specifically performed in stroke cohorts. Evidence from animal models suggest that possible neuroprotective mechanisms include activation of the cAMP/PKA pathway to promote synaptic growth and repair, reduction of blood–brain barrier leakage, and regulation of neurotransmitter transmission across synapses (120). Further studies investigating the effectiveness of GLP-1 agonists in primary prevention of PSS are warranted.

#### 4.2.5 Eslicarbazepine acetate and perampanel

Phase II clinical trials are currently ongoing to test the efficacy of eslicarbazepine acetate (90) and perampanel (91) for primary anti-epileptogenesis post-stroke. Preliminary results of the eslicarbazepine acetate trial demonstrated a trend towards lower incidence of LPSS in the treatment group compared to the placebo group (121), but analysis results of the full trial have not yet been published.

## 5 Treatment by stroke aetiology

### 5.1 Ischaemic stroke

Studies on ischaemic stroke cohorts demonstrated that statins were associated with a decreased incidence of LPSS. In contrast, r-tPA administration did not affect the incidence of PSS according to a systematic review. Regarding secondary prophylaxis, levetiracetam was linked to a 77.1% seizure-free rate at 18 months in patients with ischaemic stroke (71), whereas a small-scale study comparing lamotrigine and carbamazepine showed better efficacy for lamotrigine (64). While the primary protective effect of statins is promising, studies directly comparing the efficacy of different ASMs in ischaemic stroke cohorts are essential for guiding secondary prophylaxis.

### 5.2 Haemorrhagic stroke

Post-stroke use of statins was associated with lower incidence of LPSS in patients with intracranial haemorrhage (105). Valproic acid did not have a significant effect in prophylaxis of EPSS and LPSS in patients with spontaneous intracerebral haemorrhage (41), and early administration of benzodiazepines was also not associated with lower incidence of seizures in patients with intracerebral haemorrhage (42). Levetiracetam was associated with a lower incidence of EPSS after supratentorial intracerebral haemorrhage in a small study (62). However, two meta-analyses concluded that seizure prophylaxis after intracerebral haemorrhage was not associated with prevention of seizures, both within 14 days of intracerebral haemorrhage onset and at longest follow-up (122, 123). A Cochrane Review in 2013 found no relevant high-quality studies investigating primary and secondary prophylaxis of seizures after subarachnoid haemorrhage (124). The current AHA guidelines do not recommend prophylactic use of ASMs after intracerebral haemorrhage in patients without evidence of seizures (125) and state that randomised evidence do not support routine prophylactic ASM use after aneurysmal SAH (126). However, they suggest that prophylactic ASM use may be reasonable to prevent seizures in patients with aneurysmal SAH if accompanied by high seizure-risk features (presence of MCA aneurysm, high clinical/radiological grade, cortical infarction, or hydrocephalus).

## 6 Conclusion

Current European and US guidelines do not recommend primary prophylaxis for post-stroke seizure with ASMs in most cases, except for consideration in aneurysmal SAH with high-risk features (Table 2). Although the guidelines diverge in their recommendations for secondary prophylaxis, higher-risk patients, such as those with recurrent or late seizures and those with haemorrhagic stroke, can be treated with ASMs.

**Table 2:** Summary of society guideline recommendations on PSS prophylaxis.

Guidelines		Primary prophylaxis	Secondary prophylaxis
European Stroke Organisation (36)		Weak recommendation against primary prophylaxis	EPSS: only a weak recommendation can be made in favour of secondary prophylaxis, and we suggest not generally employing secondary ASM prophylaxis. LPSS: employing secondary ASM prophylaxis after one unprovoked seizure needs to be considered.
American Heart Association/ American Stroke Association	Ischaemic stroke (136)	Not recommended	Recurrent seizures should be treated in a manner similar to when they occur with acute neurological conditions; ASMs should be selected based on patient characteristics
	Spontaneous intracerebral haemorrhage (125)	Should not be treated prophylactically with ASMs	Recommended to improve functional outcomes and prevent brain injury from prolonged seizures
	Aneurysmal SAH (126)	Benefit of routine administration after aneurysmal SAH not supported by evidence; prophylactic ASM use may be reasonable to prevent seizures in patients with aneurysmal SAH if accompanied by high seizure-risk features	Both EPSS and LPSS warrant longer-term antiseizure medication that should be managed in the postoperative period by a clinician who specialises in seizure management.

Regarding primary prevention of PSE, there is currently no high-level evidence to support the use of an antiseizure medication or other drugs (e.g., statins) to prevent PSE. Current evidence suggests that blood–brain barrier dysfunction is a contributor to post-stroke epileptogenesis, and agents that promote repair of the barrier, such as statins and losartan, can be beneficial in minimising epileptogenic changes (127). Lipid-lowering therapy with statins is currently a standard of care in the ischemic stroke population because of its role in secondary stroke prevention and also because of its pleiotropic effects, like blood–brain barrier stabilisation (128). In context of PSE, statins appear to be associated with a lower EPSS and PSE risk in stroke patients (103). Losartan and GLP-1 agnoists are proposed to prevent epileptogenic activities in the post-stroke patient population (119, 129, 130); however, this needs to be proven in a clinical trial setting. Ongoing drug development trials like the Perampanel for the Prevention of Post-Stroke Epilepsy (*PEPSTEP*) trial (91) and the Anti-epileptogenic Effects of Eslicarbazepine Acetate (BIA-2093-213) trial (90) will need to be tested in a subsequent phase 3 trial if the results support the use of perampanel or eslicarbazepine acetate to prevent PSE. We have proposed the potential for neuroprotective agents like activated protein C for the primary prevention of PSE, but this needs testing in clinical trials as well (131). Overall, we do not recommend the use of agents specifically for the prevention of PSE due to the lack of strong evidence but note that statins could offer secondary benefits in preventing PSE in addition to their role in lipid control. Although a small potential risk of haemorrhage could be a consideration, we do not recommend withholding statins solely for this concern as the evidence base is conflicted and the benefits outweigh the risks.

Regarding secondary prophylaxis in the PSS population, a recent network meta-analysis revealed weak evidence regarding the choice of ASMs (66). Most of the evidence suggested that some second and third-generation ASMs, such as levetiracetam, lamotrigine, eslicarbazepine acetate, and lacosamide, could be effective with favourable tolerability profiles for secondary prophylaxis of PSS. First-generation ASMs, such as valproic acid and phenytoin, appeared less efficacious and had more adverse effects (66). Based on the limited available evidence, we cautiously suggest that levetiracetam and lamotrigine are preferred agents as they offer a balance of efficacy and tolerability, with the caveat that levetiracetam can affect cognition and mood, and post-stroke populations are already at risk of neuropsychiatric complications. First-generation ASMs have less favourable efficacy and risk profiles and are less preferable for PSE treatment, as was demonstrated in prospective study (132). While preliminary results on third-generation agents are encouraging, larger studies are needed to confirm their efficacy and tolerability.

Overall, the quality of much of the available evidence was low, with only three randomised control trials investigating the efficacy of ASMs on PSS primary prophylaxis or secondary prophylaxis. Currently available evidence overwhelmingly relies on retrospective analyses which may introduce publication selection bias which limits the validity of the validity of the conclusions. Inconsistent outcome measures, lack of stratification on seizure timing or stroke aetiology, and variable study designs were factors which complicated accurate head-to-head comparisons. In addition, noting the differences in the pathophysiology of EPSS and LPSS as well as between haemorrhagic and ischaemic stroke, it is likely that studies with mixed cohort can obscure the treatment effects in each group and introduce heterogeneity bias. Further studies should assess the efficacy of various ASMs stratifying for EPSS and LPSS, as well as by stroke subtype, using standardised outcomes.

In addition, the generation of seizures is multifactorial, with fluctuations in seizure threshold, seizure abnormalities, and the presence of precipitating factors (31). This often makes it difficult to predict the development and cure of epilepsy, making clinical trials of epilepsy prohibitively expensive. Risk stratification scores such as SeLECT 2.0 (24) and CAVE (19), as well as polygenic risk scores (133), aid in the selection of patients who are likely to benefit from ASM treatment. We posit that the development of effective biomarkers to select target populations and to assess their cure or remission is a priority in improving the feasibility of future clinical trials to increase the evidence base for PSS pharmacotherapy (134, 135).
